# IMPLEMENTATION & SUSTAINABILITY OF AGETECH TO SUPPORT AGING IN PLACE: PRACTICAL RECOMMENDATIONS FOR POLICYMAKERS

**DOI:** 10.1093/geroni/igad104.3419

**Published:** 2023-12-21

**Authors:** Heather McNeil, Courtney Genge, Shannon Freeman, Patricia Debergue

**Affiliations:** National Research Council, Ottawa, Ontario, Canada; National Research Council, Ottawa, Ontario, Canada; University of Northern British Columbia, Prince George, British Columbia, Canada; National Research Council, Boucherville, Quebec, Canada

## Abstract

AgeTech, a subset of the health technology industry, uses technology to support healthy aging, and support care partners and health professionals to improve quality of life for aging adults. By enhancing and adapting alternative care approaches through emerging technologies, it is possible to enable and extend the ability for older adults to safely age in place within their own homes, improve care experiences, and/or decrease long-term care costs/needs. With the rapid development and proliferation of AgeTech into the consumer market, it is paramount for policymakers in the health system to ensure that AgeTech solutions can be leveraged to support older adults to age well in place. This poster highlights five key messages for policymakers. First, AgeTech should solve a real problem. Technology must be a solution that complements care of older adults, not complicates it. Second, it is essential to embrace a life course perspective on aging, recognizing the heterogeneity of older adults who experience diverse and evolving needs. AgeTech should adapt as needs and capacities evolve. Third, health related AgeTech should empower, enhance, or support existing health care services, while recognizing the value of human interactions. In-person interactions can provide meaningful connection and important health data which should be enhanced not replaced. Fourth, the establishment and ongoing fostering of authentic partnerships to inform, co-create and co-design AgeTech solutions is key to developing successful products. Finally, policymakers and funders have an important role to play in enabling accelerated design, development and testing to meet current and future needs.

